# Liquid chromatographic nanofractionation with parallel mass spectrometric detection for the screening of plasmin inhibitors and (metallo)proteinases in snake venoms

**DOI:** 10.1007/s00216-018-1253-x

**Published:** 2018-08-09

**Authors:** Barbara M. Zietek, Morwarid Mayar, Julien Slagboom, Ben Bruyneel, Freek J. Vonk, Govert W. Somsen, Nicholas R. Casewell, Jeroen Kool

**Affiliations:** 1 Division of BioAnalytical Chemistry, Amsterdam Institute of Molecules, Medicines and Systems, Vrije Universiteit Amsterdam, de Boelelaan 1085, 1081 HV Amsterdam, The Netherlands; 2 Naturalis Biodiversity Center, 2333 CR Leiden, The Netherlands; 3 Alistair Reid Venom Research Unit, Parasitology Department, Liverpool School of Tropical Medicine, Liverpool, L3 5QA UK; 4 Research Centre for Drugs and Diagnostics, Liverpool School of Tropical Medicine, Liverpool, L3 5QA UK

**Keywords:** Plasmin, Metalloproteinase, Nanofractionation, Mass spectrometry, Snake venoms, Bioassay

## Abstract

To better understand envenoming and to facilitate the development of new therapies for snakebite victims, rapid, sensitive, and robust methods for assessing the toxicity of individual venom proteins are required. Metalloproteinases comprise a major protein family responsible for many aspects of venom-induced haemotoxicity including coagulopathy, one of the most devastating effects of snake envenomation, and is characterized by fibrinogen depletion. Snake venoms are also known to contain anti-fibrinolytic agents with therapeutic potential, which makes them a good source of new plasmin inhibitors. The protease plasmin degrades fibrin clots, and changes in its activity can lead to life-threatening levels of fibrinolysis. Here, we present a methodology for the screening of plasmin inhibitors in snake venoms and the simultaneous assessment of general venom protease activity. Venom is first chromatographically separated followed by column effluent collection onto a 384-well plate using nanofractionation. Via a post-column split, mass spectrometry (MS) analysis of the effluent is performed in parallel. The nanofractionated venoms are exposed to a plasmin bioassay, and the resulting bioassay activity chromatograms are correlated to the MS data. To study observed proteolytic activity of venoms in more detail, venom fractions were exposed to variants of the plasmin bioassay in which the assay mixture was enriched with zinc or calcium ions, or the chelating agents EDTA or 1,10-phenanthroline were added. The plasmin activity screening system was applied to snake venoms and successfully detected compounds exhibiting antiplasmin (anti-fibrinolytic) activities in the venom of *Daboia russelii*, and metal-dependent proteases in the venom of *Crotalus basiliscus*.

**Electronic supplementary material:**

The online version of this article (10.1007/s00216-018-1253-x) contains supplementary material, which is available to authorized users.

**Graphical abstract:**
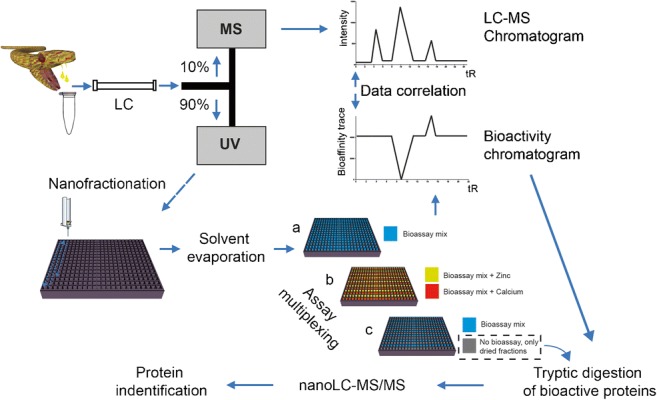
ᅟ

## Introduction

Plasmin is a trypsin-like enzyme, which plays a crucial role in the fibrinolytic pathway where it hydrolyses blood clots and maintains normal hemostasis. Plasmin originates from plasminogen, and is activated by tissue plasminogen activator (tPA) or urokinase plasminogen activator (uPA) [1]. Although the fibrinolytic activity of plasmin is regulated by the physiological inhibitors α_2_-antiplasmin and α_2_-macroglobulin, in many cases (e.g., major surgeries, hemophilia, von Willebrand syndrome, heavy menstrual bleeding), medicinal exogenous inhibitors are required to properly control its activity [1, 2]. Examples of plasmin inhibitors currently used successfully in the clinic are ε-aminocaproic acid (EACA) and tranexamic acid (TXA). However, their potency and selectivity are low, and consequently, these drugs have to be administered in high doses, which significantly increases the risk of adverse effects [1]. Therefore, there is a high demand for new, more potent and selective, anti-fibrinolytic agents.

Many snake venoms are a rich source of molecules that interfere with coagulation and fibrinolysis pathways in the hemostatic system [3]. Both anti- and pro-fibrinolytic activities have been observed, mostly in the venoms of the *Viperidae* snake family [4]. Antiplasmin proteins usually belong to the Kunitz/bovine pancreatic trypsin inhibitors (BPTI) family and so far a number of Kunitz-type protease inhibitors have been isolated and purified from snake venoms, including Kunitz inhibitor-I (DrKIn-I) and -II (DrKIn-II) from *Daboia russelii* [5], and the textilinins from *Pseudonaja textilis* [6].

The fibrin(ogen)olytic effect of snake venom toxins results from different mechanisms, e.g., stimulation of plasminogen activators from endothelial cells [7], direct plasminogen activation [8, 9], or direct cleavage and degradation of fibrinogen and fibrin [10–13]. Most of the molecules which exhibit fibrin(ogen)olytic activities belong to the snake venom serine protease (SVSP) and snake venom metalloproteinase (SVMP) toxin families [4, 14]. Both SVSPs and SVMPs are proteolytic enzymes of which the latter require zinc ions as metal co-factors for catalytic activity, and in some cases calcium for structural stability [14–16].

SVMPs have been recognized as one of the main components of venoms responsible for causing pathologies associated with snakebites [15]. Snakebites remain an important and neglected public health issue [17] especially in poorer, tropical areas of the world where access to healthcare is problematic. Such bites result in extensive mortality (~ 94,000 deaths/annum) and morbidity (~ 3–5 times the number of annual deaths), as the result of the hemorrhagic, coagulopathic, neurotoxic, and/or cytolytic effect of different snake venoms [18, 19]. Apart from their high hemorrhagic activities, which is most profound in the P-III class, SVMPs are reported to exhibit fibrin(ogen)olytic, apoptotic, inflammatory, and factor Xa and prothrombin-activating activities [14]. Considering the significant hazard that SVMPs represent to victims of envenomation, information on their presence, molecular structure and bioactive properties will help in the development of more specific, and thus more effective treatments for snake envenoming, such as aptamers [20], snake venom toxin inhibitors [21, 22], recombinant anti-venoms [23] or chelating agents [22, 24].

Due to the complex nature of snake venoms, multiple analytical techniques are required for the identification and biochemical characterization of individual toxins. A straightforward and effective methodology for screening venoms for selected bioactivities is nanofractionation analytics [25–27]. In this approach, a venom is first separated by liquid chromatography (LC) after which the column effluent is split, with one part being fractionated with high resolution into a high-density microtiter well plate (typically 96 to 1536 wells) and the other part being interrogated simultaneously by mass spectrometry (MS) analysis. The collected fractions on the microtiter plate are subsequently exposed to a bioassay. As chromatographic resolution is essentially maintained, the results of the bioassay readout can be constructed into a bioactivity chromatogram, which can be accurately correlated with the parallel obtained MS chromatogram. Besides venom research, the nanofractionation technique also has shown to be useful for drug-drug interaction profiling [23], environmental analysis [28], and profiling natural extracts [29].

In this study, we present nanofractionation analytics for the screening of plasmin inhibitors and proteases. We applied the developed methodology to the analysis of venoms from the medically relevant viperid snakes *Daboia russelii* and *Crotalus basiliscus* for the presence of plasmin inhibitors as well as (metallo)proteinases that exhibit fibrin(ogen)olytic activity similar to plasmin. *Daboia russelii,* also known as Russell’s viper*,* is responsible for substantial proportion of snakebite-induced morbidity and mortality in Asia, and causes diverse symptoms such as coagulopathy, swelling, neurotoxicity and pain [30]. *Crotalus basiliscus*, known as the Mexican west-coast rattlesnake, as indicated by its name can be found mainly in Mexico. Envenomation by this snake leads to local tissue damage, systemic bleeding and hypotension [31]. The fibrin(ogen)olytic proteins were further evaluated for their activity dependency on metal ions by applying to the nanofractionated venoms the plasmin bioassay mixture enriched with zinc and calcium ions, and also in the presence of the metal chelating agents 1,10-phenanthroline or EDTA. Plasmin inhibitors and proteolytic enzymes detected by the applied screening methodology were subsequently characterized.

## Material and methods

### Chemicals and biological samples

Human plasmin was purchased from Haematologic Technologies, Inc. (Essex Junction, VT, USA) and used at 670 μg/mL in 1:1 glycerol/water solutions (*v*/*v*). Fluorogenic substrate H-D-Val-Leu-Lys-AMC was purchased from BACHEM (Bubendorf, Switzerland) and dissolved in DMSO to a stock concentration of 40 mM. Leupeptin hydrochloride (dissolved in Milli-Q water to obtain a stock of 10 mM), 1,10-phenanthroline, bovine serum albumin (BSA), calcium chloride_,_ DMSO, EDTA, iodoacetamide, trypsin from bovine pancreas, and Trizma were all obtained from Sigma (Zwijndrecht, The Netherlands). Hydrochloric acid (HCl) was received from Riedel-de-Haën (Zwijndrecht, The Netherlands), formic acid (FA) and β-mercaptoethanol were purchased from Merck (Darmstadt, Germany), and ULC-MS grade acetonitrile (ACN) was obtained from Biosolve (Valkenswaard, The Netherlands). Lyophilized snake venoms were from *Daboia russelii* (*Dr*) and *Crotalus basiliscus* (*Cb*), which both are from the *Viperidae* family. The *Daboia russelii* venom (Sri Lankan origin) was provided by Dr. Nicholas Casewell from the Alistair Reid Venom Research Unit, Liverpool School of Tropical Medicine, and the *Crotalus basiliscus* venom (Captive bred), was provided by Dr. Freek Vonk, Naturalis Biodiversity Center. Depending on the experiment performed, solutions of the venoms were prepared in Milli-Q water at concentrations of 4 mg/ml, 5 mg/mL or 0.5 mg/ml and stored at − 80 °C.

### LC, nanofractionation and MS detection

Bioassay optimization and analyses of crude snake venoms were performed using a Shimadzu high-performance liquid chromatography (HPLC) system (‘s Hertogenbosch, The Netherlands) consisting of a Shimadzu SIL-30 AC autosampler and a Shimadzu LC-20AB binary pump, which were interfaced to an Ultima Q-TOF MS instrument (Waters, Bradfrod, UK). *Dr* and *Cb* venoms at a concentration 4 mg/ml (injected volume, 45 μL) and leupeptin standards (injected volume, 20 μL) at five different concentrations (ranging from 6 to 200 μM) were separated with a Waters XBridge C18 column (4.6 × 100 mm, particle size 5 μm) (Milford, MA). The analytical column was protected by a guard column comprising the same packing material. Both columns were kept in a CTD-30 column oven (Shimadzu) set to 37 °C. Gradient elution was performed employing mobile phase A (98% water, 2% ACN, 0.1% FA (*v*/*v*/*v*)) and mobile phase B (98% ACN, 2% water, 0.1% FA (*v*/*v*/*v*)). The gradient used for separation started at 0% B with a linear increase to 50% B in 20 min followed by an increase to 90% B in 2 min. The gradient was held at 90% B for 2 min, and then returned in 1 min to the starting conditions (0% B). Column re-equilibration was 5 min, resulting in a total analysis time of 30 min. The flow rate was 0.5 ml/min. Using a post-column flow split, 90% of the eluate was directed via a Shimadzu SPD-20A UV detector (set at 200 nm) to an in-house developed nanofractionation system, and the other 10% of the eluate was analyzed with an Ultima Q-TOF mass spectrometer equipped with electrospray ionization in positive ion mode (ESI^+^). The following settings of the ionization source were used: capillary voltage, 3.25 kV; source temperature, 125 °C; desolvation temperature, 200 °C; gas flow, 250 L/h. The mass range was set to 50–2000 *m/*z.

Detailed information on the nanofractionation system can be found elsewhere [28, 32]. Briefly, the system was built from a modified 235 Gilson autosampler, in which a fused silica capillary extension connected to LC tubing made of polyether ether ketone (PEEK) was mounted to the robotic arm, which allowed the capillary needle to move in xy directions. Fractions of 6 s were collected onto a black 384-well plate (Greiner Bio One, Alphen aan den Rijn, The Netherlands) in a serpentine way. After nanofractionation, the plates were evaporated overnight (O/N) using a RVC 2–33 CD plus maxi concentrator (Salm en Kipp, Breukelen, The Netherlands) with a rotation speed of 1500 rpm, pressure of 0.10 mbar, and temperature of 30 °C. After solvent evaporation, the dried plates were stored at − 20 °C prior to bioassaying. A simplified scheme showing of the analytical method can be found in Electronic Supplementary Material (ESM) Fig. S1.

### Plasmin bioassay

Screening for snake venom components modifying the activity of plasmin was performed with an optimized bioassay mixture containing 100 ng/mL plasmin and 5 μM fluorogenic substrate H-D-Val-Leu-Lys-AMC dissolved in 100 mM TRIS-HCl buffer (pH 7.5) containing 0.1% BSA (*w*/*v*). The bioassay optimization results can be found in the ESM Section S1.1. The bioassay mixture was prepared by adding equal volumes of substrate and the enzyme solutions in 100 mM Tris-HCl buffer with 0.1% BSA. The buffer solution was at room temperature (RT), whereas the enzyme and substrate stock solutions were kept at − 20 °C. Prior to bioassaying, the enzyme and the substrate were thawed and diluted in buffer contained in two Greiner tubes. The solutions were carefully mixed (for 5 s) to prevent air bubble formation. Directly after preparing 50 μL of the bioassay mixture was pipetted over a black 384-well plate (Greiner Bio One, Alphen aan den Rijn, The Netherlands) using a Multidrop 384 reagent dispenser (Thermo Scientific, Ermelo, The Netherlands). A VarioSkan LUX microplate multimode reader (Thermo Scientific, Ermelo, The Netherlands) was used to measure fluorescence of each well kinetically at 380 and 460 nm excitation and emission wavelength, respectively. The temperature inside the plate reader was set at 37 °C. Each measurement comprised 10 cycles allowing generation of a kinetic curve, and subsequent determination of its slope, which was plotted against the time of nanofraction collection, producing a so-called bioactivity chromatogram. Negative peaks in the bioactivity trace represent inhibition of plasmin, and positive peaks indicate the presence of plasmin inducers or, more likely in the case of venoms, proteases exhibiting an enzymatic activity similar to plasmin.

The *Z*′ factor, introduced by Zhang et al. [33], was used as statistical parameter to assess the quality of the bioassay. For that, the inhibitor leupeptin (200 μM concentration representing full enzyme inhibition) and a sample containing mobile phase A were used as a positive and negative control, respectively. Both solutions were pipetted over a 384-well plate at volumes of 10 μL per well using the Multidrop 384 reagent dispenser. Next, the plates were vacuum-centrifuge evaporated and exposed to the plasmin bioassay. Measurements were performed in duplicate.

### Bioactive compound identification and sample purification for proteomics

#### Reversed-phase (RP) LC and hydrophilic interaction liquid chromatography (HILIC) orthogonal separation strategy, fraction collection, proteomics

Identification of bioactive compounds from venoms of *Dr* and *Cb* involved nanofractionation onto 384-well plates with parallel high-resolution MS analysis using a Maxis HD quadrupole time-of-flight MS (Bruker Daltonics, Bremen, Germany). For nanofractionation and MS, 5 mg/ml *Dr* venom and 0.5 mg/ml *Cb* venom (50 μL injected using a SIL-20AC autoinjector; Shimadzu, Canby, OR, USA) were separated on an XBridge BEH300 C18 (4.6 × 150 mm; particle size, 3.5 μm) analytical column placed in a column oven (model CTO-20AC Shimadzu, Canby, OR, USA) kept at 30 °C. A binary mobile phase consisting of mixtures of solvent A (98% water, 2% ACN, 0.1% FA (*v*/*v*/*v*)) and solvent B (98% ACN, 2% water, 0.1% FA (*v*/*v*/*v*)) was used for gradient elution: 0–50% B in 20 min, 50–90% B from 20 to 24 min, isocratic at 90% B from 24 to 29 min, back to 0% B in 0.1 min. Column equilibration was then performed for another 10 min using 0% B, resulting in a total analysis time of 40 min. The flow rate was 0.5 ml/min The MS was equipped with an ESI source (Apollo) used in positive mode. The capillary voltage was 4.5 kV, and nebulization was done with nitrogen gas (0.4 bar). Ion desolvation was performed using nitrogen as a drying gas heated to 200 °C and flowing at a rate of 4 l/min. Mass accuracy was assured by internal calibration using an ESI-L low concentration tune mix (Agilent, Santa Clara, California, USA) in every run.

An orthogonal separation of the two crude venoms was performed using an Atlantis silica HILIC (4.6 × 150 mm; pore size, 100 Å; particle size, 3 μm) analytical column. The separation was performed using gradient elution with solvent A composed of 98% ACN, 2% water, 0.2% FA (*v*/*v*/*v*) and solvent B of 98% water, 2% ACN, 0.2% FA (*v*/*v*/*v*). The gradient started at 30% B and increased to 50% B in 15 min. The next 10 min, the gradient rose to 95% B and remained at 95% B for 10 min. Within 2 min, the gradient returned to initial conditions. Column equilibration was set for 23 min at 30% B resulting in a total analysis time of 60 min. The flow rate was 0.5 ml/min. MS conditions were the same as used for RPLC analysis.

The nanofractionated venoms were exposed to the plasmin bioassay by robotic pipetting of the bioassay mixture to every second well on the plate. As an eluting compound usually is distributed over multiple wells, the alternating wells (i.e., without bioassay mixture) could be used for other analyses (e.g., proteomics). From the bioactivity chromatograms, wells with bioactive compounds were identified and the content of adjacent wells was subsequently subjected to proteomic analysis. The content was dissolved in 100 μL water and after 30 min, 50 μL samples were transferred to low-protein binding Eppendorf tubes containing digestion buffer and reduction agent for tryptic digestion (see “Tryptic digestion of bioactive proteins” section).

Throughout the study, leupeptin was used as a time calibration point for accurate correlation of the bioactivity and MS chromatograms. Leupeptin was analyzed using RPLC and HILIC separation (ESM Figs. S2A and S2B, respectively). This experiment enabled calculation of the time delay, which is observed in the MS data acquisition compared to the nanofractionation process. This delay is due to differences in LC-tubing length between the two systems, the column used and the flow rates applied, and has to be measured after each change made in the system.

#### Tryptic digestion of bioactive proteins

Bioactive proteins present in the venoms analyzed underwent tryptic digestions according the following protocol. Briefly, reduction of disulfide bonds was performed by adding 50 μL of each bioactive fraction (reconstituted in 100 μL water) from a nanofractionated snake venom to a solution containing 75 μL of 25 mM ammonium bicarbonate (pH 8.2) and 7.5 μL of 0.5% β-mercaptoethanol prepared in water (*v*/*v*). The solutions were shortly vortexed and then incubated at 95 °C for 10 min using a dry block heating thermostat (Bio TDB-100; Biosan Ltd., Riga, Lativa). After cooling down to room temperature, the solutions were centrifuged at a speed of 21,500 rpm for 10 s. Next, the reduced proteins were alkylated for 30 min with 15 μL of 100 mM iodoacetamide in the dark at room temperature. After the alkylation procedure, proteins were digested by adding 3 μL of a 0.1 μg/μL trypsin solution prepared in 25 mM ammonium bicarbonate (pH 8.2). The stock solution of the trypsin used was 1 mg/ml prepared in 1 mM HCl and stored at − 20 °C. The samples were incubated for 3 h at 37 °C followed by addition of an extra 3 μL of the same trypsin solution and then incubated O/N at 37 °C. The digestion was quenched with 5 μL of 5% formic acid in water (*v*/*v*) followed by centrifugation at 21,500 rpm for 10 s. The collected supernatant was transferred to LC-MS vials and analyzed with nanoLC-ESI-MS/MS (see “NanoLC-MS/MS analysis of the tryptic digests” section).

#### NanoLC-MS/MS analysis of the tryptic digests

Following tryptic digestion, samples were separated and analyzed with an Ultimate 3000 Nano HPLC system (Thermo Scientific) interfaced to a Bruker Maxis quadrupole time-of-flight MS instrument. Detailed information on these analyses can be found in the ESM Section S1.2. Identification of proteins was performed with the search engine Mascot (version 2.301). Raw MS data were converted into centroided peak lists using DataAnalysis version 5.0 (Bruker). The parameters used for Mascot searches were set as follows: enzyme specificity, semiTrypsin; missed cleavages, 2; fixed modification, carbamidomethyl (C); variable modification, oxidation (M) peptide mass tolerance, 0.1%; fragment mass tolerance, 0.05 Da; no restriction for species was used. The instrument was set to ESI-QUAD-TOF.

## Results and discussion

### Evaluation of the plasmin bioassay

Screening snake venoms for plasmin inhibitors and (metallo)proteinases exhibiting activity similar to plasmin was performed with a fluorescence based bioassay based on the protocol reported by Tervo et al. [34]. Bioassay optimization is described in the ESM section S1.1 and Fig. S3. The optimized bioassay mixture comprised 100 ng/mL end concentration of plasmin enzyme and fluorogenic aminocoumarin-based substrate H-D-Val-Leu-Lys-AMC at a final concentration of 5 μM, in 100 mM Tris-HCl buffer (pH 7.5) containing 0.1% BSA. BSA was used following the findings by Tervo et al. [34], who observed that it prolonged the enzymatic activity of plasmin. In our study, 0.1% BSA increased the signal-to-noise ratio (S/N) approximately three times and improved the baseline stability (data not shown).

**Fig. 1:**
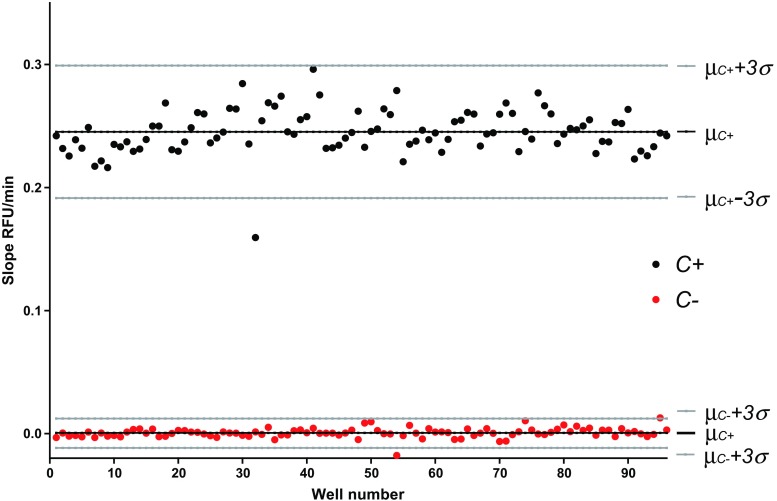
Assessment of the bioassay performance. The *Z*′-factor for the bioassay was calculated to be 0.74. Positive control (*C+*) wells contain mobile phase A (*n* = 96); negative control (*C−*) wells contain 200 μM leupeptin (*n* = 96). The wells were evaporated to dryness prior to the bioassay

A well-performing bioassay should have a statistical *Z*′ factor between 0.5 and 1 [33]. To determine the *Z*′ factor for the plasmin bioassay, the plasmin inhibitor leupeptin at 200 μM (causing full inhibition of the enzyme) and a sample containing mobile phase A were used as positive and negative controls, respectively (Fig. 1). The *Z*′ value for the plasmin bioassay was found to be 0.74, which is considered excellent. The obtained S/N at maximum inhibition of plasmin was high with an average S/N of 54.

### Optimization and validation of the nanofractionation approach

**Fig. 2:**
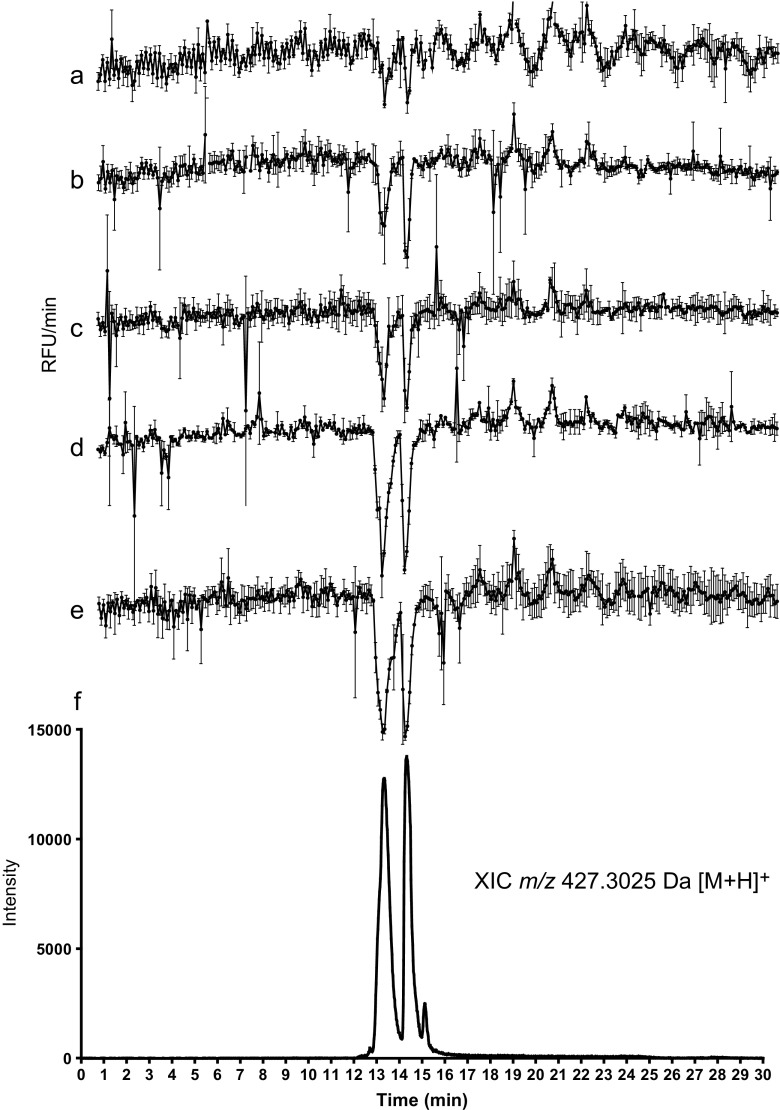
Evaluation of the overall performance of the nanofractionation system using the plasmin inhibitor leupeptin, which was nanofractionated at five concentrations injected. (**a**) 6.3 μM. (**b**) 25 μM. (**c**) 50 μM. (**d**) 100 μM. (**e**) 200 μM. (**f**) The extracted ion chromatogram (*m/z* 427.3, [M + H]^+^) of the analysis of 100 μM leupeptin with online LC-MS. RFU, relative fluorescence units

To evaluate the performance of the total nanofractionation/bioassay system, the plasmin inhibitor leupeptin was analyzed in different concentrations. The experimentally determined IC50 for leupeptin was 8.4 μM (data not shown). Figure 2 shows bioactivity chromatograms of the different leupeptin solutions analyzed, which correlated nicely with the parallel recorded MS data, as shown for the analysis of 100 μM leupeptin, detected at *m/z* 427.3. The bioactivity chromatograms show inhibition of plasmin as negative peaks. Leupeptin shows multiple peaks in the XIC at 13.3 and 14.3 min as it comprises a mixture of diasteroisomers of leupeptin that are generated in solution due to racemization reactions [35]. The two peaks at 13.3 and 14.3 min are seen in the bioactivity chromatograms. The peak shapes of the two diastereoisomeric peaks in the bioactivity trace could exactly be correlated by the parallel obtained MS chromatogram. The sigmoidal concentration–response relation of the bioassay causes the bioactivity peaks to show broadening at high concentrations leupeptin injected, which is quite common for this type of analyses [26, 36]. Chromatographic band dispersion, spreading of eluting compounds over multiple wells and additional dilution of the dried wells after reconstitution of the deposited components in the wells with bioassay mixture, results in a difference between bioassay concentration and injected concentration, denoted as dilution factor [37], which was ~ 3.2.

### Profiling plasmin inhibition and proteolytic activity in *Cb* and *Dr* venoms

**Fig. 3:**
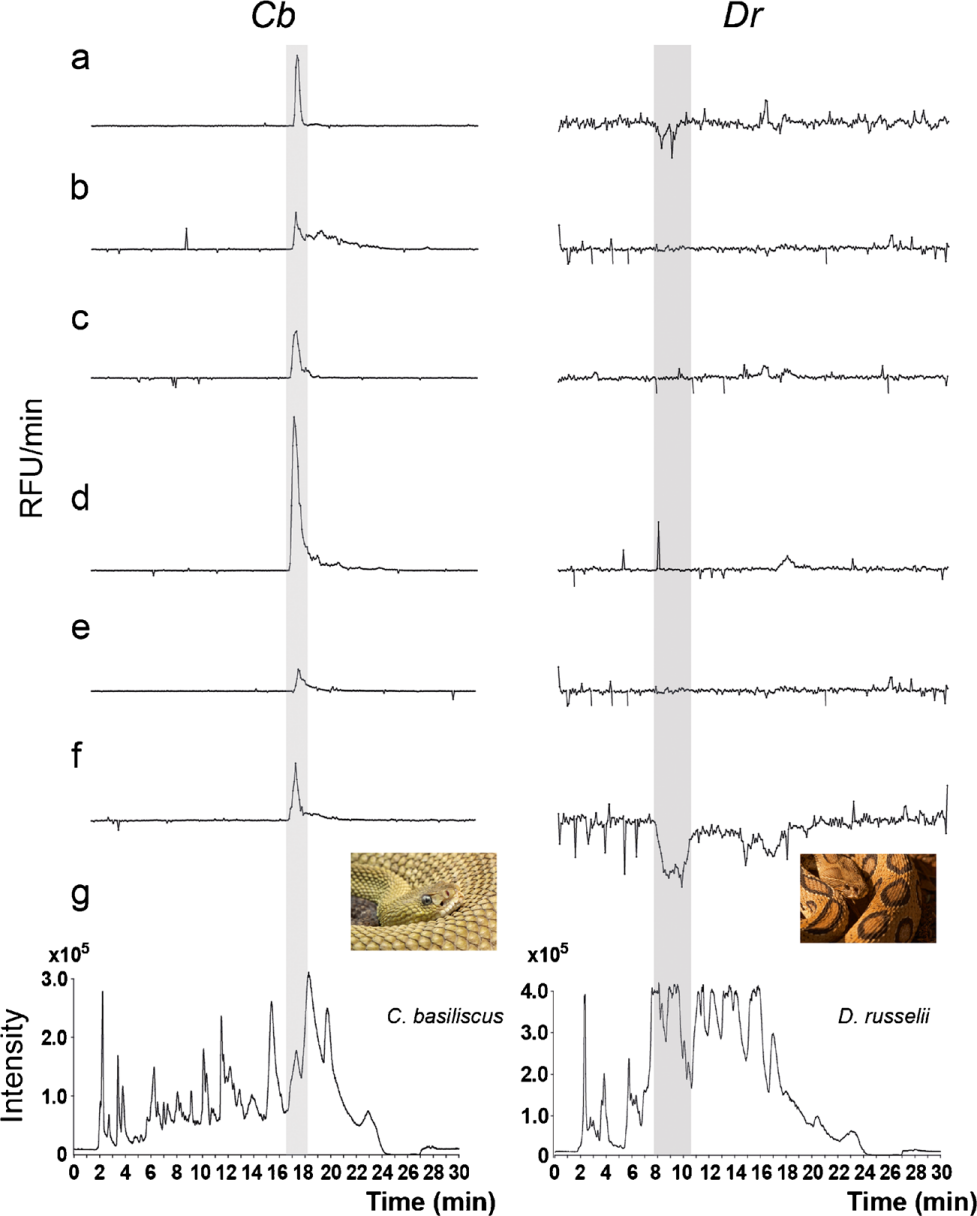
(**a**–**f**) Bioactivity chromatograms obtained for *Cb* and *Dr* venoms using the following assay variants. (**a**) Full plasmin bioassay mixture containing plasmin and substrate. (**b**) Bioassay mixture containing only the substrate. (**c**) Bioassay mixture containing only the substrate and buffer enriched with zinc ions. (**d**) Bioassay mixture containing only the substrate and buffer enriched with calcium ions. (**e**) Bioassay mixture containing only the substrate and buffer enriched with EDTA. (**f**) Bioassay mixture containing plasmin, substrate and 1,10-phenanthroline. (**g**) Total-ion chromatogram obtained for *Cb* and *Dr* venoms with online parallel LC-MS. The rectangular gray shades indicate the elution of the bioactive peaks. Photos of the snakes: *Crotalus basiliscus* (source: Shutterstock/Bernhard Richter); *Daboia russelii* (source: Shutterstock/Meet Poddar). RFU, relative fluorescence unit

Venoms of *Cb* and *Dr* were analyzed for (1) the presence of proteins exhibiting inhibitory activity towards plasmin, and (2) the presence of proteases exhibiting plasmin-like activities and/or plasmin activators. Figure 3 shows results obtained after injection of 4 mg/ml of the venoms. In the bioactivity chromatograms of *Cb* venom (Fig. 3a), no negative peaks were observed implying that *Cb* venom did not contain components with significant antiplasmin (anti-fibrinolytic) activity. To our knowledge, no anti-fibrinolytic activities of *Cb* venom have been reported in the literature. However, the bioactivity chromatograms of *Cb* venom showed a clear positive peak between 17 and 18 min, indicating protein components with possible proteolytic and fibrin(ogen)olytic activity. *Cb* venom is known to have fibrinolytic properties and a number of fibrinolytic enzymes have previously been isolated from this venom [31, 38, 39]. To investigate whether the positive peak observed resulted from proteases exhibiting proteolytic properties similar to plasmin, or resulted from a plasmin activity inducer, variants of the bioassay were applied (see below).

The analysis of the venom of *Dr* resulted in bioactivity chromatograms showing clear inhibition of plasmin (Fig. 3a). Injection of a venom sample of 4 mg/ml showed multiple negative peaks eluting at 8.9 and 9.9 min, clearly indicating the presence of antiplasmin/anti-fibrinolytic activity. *Dr* venom has previously been reported to contain the Kunitz-type protease inhibitors DrKIn-I and -II which exert anti-fibrinolytic activity. Some reports, however, showed that *Dr* venoms also contain SVSPs and SVMPs, which could potentially exert fibrin(ogen)olytic properties [40, 41]. However, in our study, positive peaks were not observed.

For further investigation of the increased enzyme activities observed for the two venoms, six different variants of the plasmin bioassay were applied. In the first variant, the bioassay mixture was prepared without the addition of plasmin to test whether the active protein is a plasmin analogue that converts similar substrates or is a plasmin activator. In the second and third variants, the bioassay mixture was prepared without plasmin and was enriched with calcium (1 mM) or zinc (0.2 mM) ions, respectively, to determine whether bioactivity could be ascribed to a metalloproteinase and, if so, to differentiate between calcium or zinc dependency. To confirm that the resulting bioactive protein is a metalloproteinase, in the fourth and fifth variant of the bioassay, the metal ion chelators EDTA (50 mM) or 1,10-phenanthroline (5 mM) were added, while again excluding plasmin from the mixture. The chelator 1,10-phenanthroline specifically binds zinc ions, deactivating zinc-dependent metalloproteinases only, whereas EDTA chelates both zinc and calcium ions [42]. In the sixth and final variant, 1,10-phenanthroline was added to the full bioassay mixture, in order to deactivate SVMPs (if present) and allow the detection of inhibitors that are co-eluting with SVMPs.

Results from the analyses of both venoms using the six assay variants are shown in Fig. 3a–f for *Cb* and *Dr*, respectively. An extensive discussion of the results of these experiments can also be found in the ESM Section S2.1. Briefly, in the venom of *Cb*, the major positive peak was also observed when plasmin was not included in the bioassay mixture. This indicates that the bioactive protein detected is not a plasmin inducer, but an enzyme that is capable of converting the substrate. Subsequent experiments (bioassay variants 2–5) showed that the activity of the bioactive protein increased about threefold (compared to the results obtained with substrate only [variant 1]) when calcium ions were added to the bioassay mixture, indicating possible calcium dependency or increased stability of the protein. The addition of zinc ions did not affect the intensity of the bioactivity peak. To assure that the changes observed in the bioassay are not a result of the variation between different plates/bioassays tested, additional experiments confirming the dependence of the bioactive compounds on metal ions were performed. Two bioassay mixtures, with added calcium and zinc ions, respectively, were pipetted into alternating wells of one microtiter well plate containing nanofractionated venom. This was done to exclude potential inter-experiment variations between nanofractionated runs. The results (ESM Fig. S4a) showed an activity-inducing effect of the calcium ions on the eluted bioactive proteins, confirming the results of the earlier experiments. Figure 3 shows that, in the case of *Dr* venom, no increased enzyme activity was observed, indicating that the bioactive compounds detected (Fig. 3a–f) were predominantly plasmin inhibitors (anti-fibrinolytic agents) [43].

### Identification of the bioactives

**Fig. 4:**
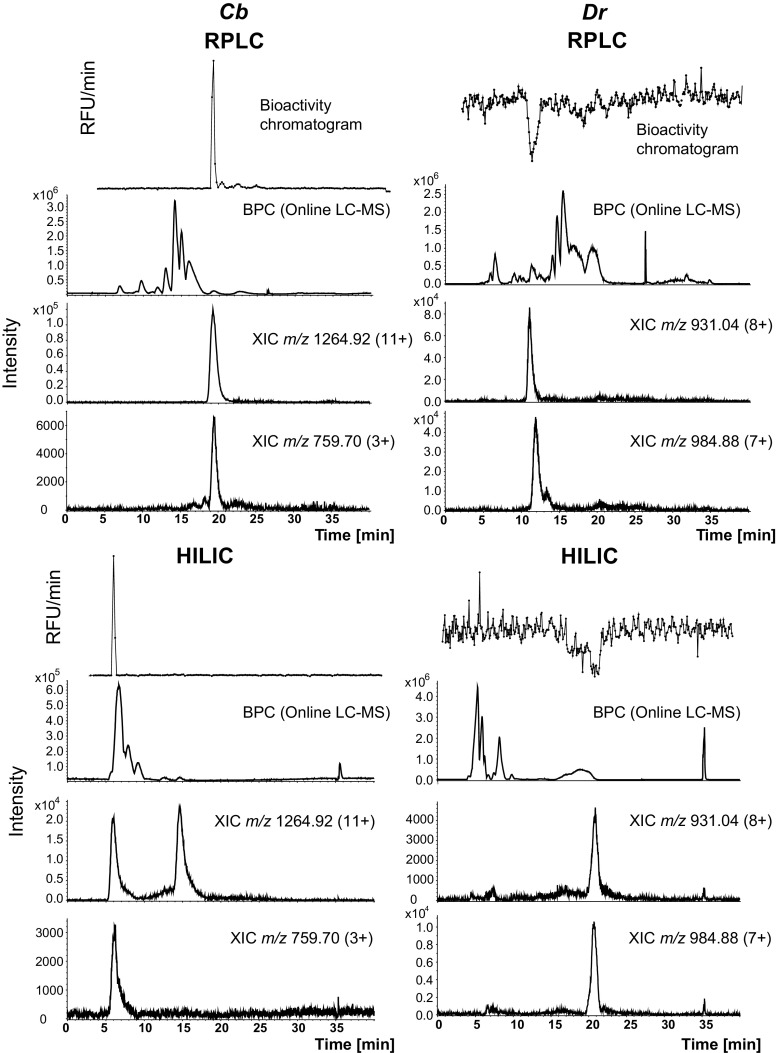
Correlation of bioactivity and MS chromatograms obtained for *Cb* (*C. basiliscus*) and *Dr* (*D. russelii*) venoms after RPLC and HILIC separations. The data were acquired online in parallel to the nanofractionation with LC-MS. XIC, extracted ion chromatogram; BPC, base peak chromatogram; RFU, relative fluorescence unit

For identification of the observed bioactive proteins, the two crude venoms were each nanofractionated twice using the orthogonal chromatographic separation mechanisms RPLC and HILIC. The resulting nanofractionated plates were then exposed to the bioassay mixture by pipetting it to the even rows, leaving the odd rows empty (containing only the dried fractions). Based on the bioactivity chromatograms resulting from these experiments, information on the elution time of the bioactives, their positions in the well plates and their molecular weight was obtained for each stationary phase used. Subsequently, wells containing dried fractions which were not exposed to the bioassay mixture, but which were adjacent to wells with bioactive proteins, were subjected to tryptic digestion and proteomic analysis. The bioactivity chromatograms correlated to corresponding MS data for *Cb* and *Dr* venoms are presented in Fig. 4. The bioactivity chromatogram for *Cb* venom (0.5 mg/ml injected concentration) analyzed with RPLC (Fig. 4) displayed a clear positive peak at 19.5 min. The HILIC analysis of *Cb* venom showed a bioactive component peak eluting as a sharp peak between 5.7 and 6.5 min. The *m/z* values that correlated with the bioactivity peak for both bioactivity chromatograms were 1264.92 (charge state + 11 protein mass 13,903.06), and 759.70 (charge state + 3; peptide mass 2276.05). Based on the earlier experiments in which different variants of the plasmin assay were used, the bioactive is expected to be a calcium-dependent protease. However, no protein with a molecular mass between 20 and 100 kDa (i.e., the mass range of known snake venom metalloproteinases) was observed, most likely due to the limited ionization efficiency achieved for larger intact proteins. The highest mass that was detected with RPLC-MS and HILIC-MS was ~ 15 kDa, which matches well with the phospholipase A_2_ (PLA_2_) family (13–15 kDa). Considering that venom is a complex mixture, some peptides and proteins will not be separated and will coelute making the determination of the bioactive peptides more difficult. The presence of the mass (15 kDa) can be explained by coelution of PLA_2_ with the real bioactive observed in both bioactivity chromatograms [3, 44]. Slightly different results were obtained from the proteomic analysis of the bioactive wells. Mascot search results obtained from the nanoLC-MS data for the trypsin-digested bioactive proteins revealed the presence of peptide sequences that matched SVSPs and SVMPs. Together, five proteins were found in the samples obtained from RP analysis, and eight proteins were found after HILIC. The peptide sequences found in the venom sample analyzed with RPLC matched the proteins catroxase-1 (protein coverage, 10%; protein score, 99), for which two distinct peptides were found, and catroxase-2 (protein coverage 17%; protein score, 91), with two distinct peptides. Both of the catroxases came from *Crotalus atrox*. A match was also found with a serine protease from *Crotalus durissus* (protein coverage, 13%; protein score, 233), for which two peptides were found. Although the Mascot report showed the two catroxases to fit into the family of SVSPs, the protein catroxase on several occasions has been reported to be a SVMP [13, 45]. For example*,* Chiou et al. [13] reported catroxase as a fibrinolytic protein that belongs to the family of SVMPs, as it was found to be inhibited by EDTA*.* This discrepancy can be explained by the fact that several catroxases exist and they are a mixture of SVSPs and SVMPs (http://www.uniprot.org/uniprot/?query=CATROXASE&sort=score). It is important to mention, however, that the sequence of SVMP catroxase has been only partially determined. It is possible that our sample contains a bioactive protein with a sequence that matched partially with SVSP cartoxases found in *C. atrox*; however, it still possesses metal-dependency, as showed by its inhibition with EDTA. This information supports our earlier results suggesting that the bioactive could be a fibrinolytic calcium-dependent metalloproteinase. In addition, we recovered peptide sequences matching SVSPs from *Protobothrops jerdonii* (protein coverage 8%, protein score 100) with a single matched peptide, and an acidic phospholipase A_2_ from *Crotalus viridis* (protein coverage 18%, protein score 59) with two peptides matched. The SVMP atrolysin-d, which is known to have calcium and zinc binding sites (http://www.uniprot.org/uniprot/P15167), was found after HILIC-MS analysis (protein coverage 3%, protein score 123), for which a single peptide was found. Detailed information obtained from the Mascot searches revealing the protein coverage and scores for *Cb* venom analyzed with RPLC and HILIC can be found in ESM Table S2.

When *Dr* venom (injected concentration, 5 mg/ml) was analyzed by RPLC-MS and HILIC-MS, the observed bioactivity peaks correlated with *m/z* 931.04 Da (charge state + 8; peptide mass 7440.25) and *m/z* 984.88 (charge state + 7; peptide mass 6887.10) (Fig. 4). Further analysis of the trypsin-digested bioactive well with nanoLC-MS and Mascot analysis obtained after RPLC revealed the presence of four proteins, and in the case of HILIC analysis, the number of found proteins was five. The matched proteins include the Kunitz-type serine protease inhibitor C6 from *Daboia siamensis* venom (protein coverage, 43%; protein score, 343) for which five peptides were found, and Kunitz-type serine protease inhibitors 3 and 4, both from *Daboia russelii* venom (protein coverage, 36%; protein score, 58, three matched peptides; and protein coverage, 33%; protein score, 125, one matched peptide, respectively). For the proteomic analysis of bioactive fractions collected after HILIC separation, the best match was for the co-eluting basic PLA_2_ VRV-PL-VIIIa (protein coverage, 87%; protein score, 2173), for which 17 peptides were found, and PLA_2_ VRV-PL-V (protein coverage, 71%; protein score, 1935), for which seven peptides were found. Both proteins were found to be from *Daboia russelii* venom. Kunitz-type serine protease inhibitor 3 was also found in the HILIC bioactive fraction, however, at a protein score of 65 and protein coverage of 20%, with a single peptide matched. Detailed results from Mascot analysis listing all protein and peptides found together with the protein and peptides scores, % coverage, observed and expected masses of the peptides can be found in ESM Table S3.

## Conclusions

In this study, a nanofractionation platform was developed and applied for the screening of snake venoms for plasmin inhibitors and (metallo)proteinases with similar activity to plasmin. A 384-well format plate reader fluorescence assay for plasmin activity was optimized and implemented in the platform. The usefulness of the methodology was demonstrated by analyzing venoms of the snakes *Daboia russelii* and *Crotalus basiliscus*. In *Dr* venom, plasmin inhibitors were detected and identified by downstream proteomic analysis. In *Cb* venom, fibrin(ogen)olytic enzymes that act in a manner similar to plasmin were found. These enzymes were shown to be potentially calcium dependent proteases that exhibited similarity to known SVMP venom toxins. The method developed is a reliable and high-throughput screening tool that helps in discovering new drug leads by exposing individual proteins present in snake venoms to a bioassay mixture containing a drug target of interest. The parallel MS analysis and off-line proteomic studies that follow the bioactivity assessment allow for bioactive protein identification in a high-throughput manner. Understanding the composition of snake venoms, in particular toxins that are responsible for the life debilitating and life-threatening manifestation of snakebites, may also aid in the development of new toxin-specific anti-venoms that have already been demonstrated to have the potential to neutralize toxins in venoms of snakes species that are geographically and phylogenetically distinct [22].

## Electronic supplementary material

ESM 1 (PDF 1.01 MB)
