# Supplementary material for: Liquid chromatographic nanofractionation with parallel mass spectrometric detection for the screening of plasmin inhibitors and (metallo)proteinases in snake venoms
Source: Anal Bioanal Chem. 2018 Aug 9;410(23):5751–63. doi: 10.1007/s00216-018-1253-x (PMC6096707; doi:10.1007/s00216-018-1253-x)
Supplement: Supplementary file 1 — (PDF 1.01 MB) [file 216_2018_1253_MOESM1_ESM.pdf]

Analytical and Bioanalytical Chemistry

Supplementary Materials

Supplementary Figures S1-S4 and Tables can be found below the text

Liquid chromatographic nanofractionation with parallel mass spectrometric detection for the screening of plasmin inhibitors and (metallo)proteinases in snake venoms

Barbara M. Zietek[1], Morwarid Mayar[1], Julien Slagboom[1], Ben Bruyneel[1], Freek J. Vonk[2] Govert W. Somsen[1], Nicholas R. Casewell[3, 4], Jeroen Kool[1*]

*Corresponding author: Jeroen Kool, j.kool@vu.nl

[1] *Division of BioAnalytical Chemistry, Amsterdam Institute of Molecules, Medicines and Systems, Vrije Universiteit Amsterdam, de Boelelaan 1085, 1081 HV Amsterdam, The Netherlands*

[2] *Naturalis Biodiversity Center, 2333 CR Leiden, The Netherland*

[3] *Alistair Reid Venom Research Unit, Parasitology Department, Liverpool School of Tropical Medicine, Liverpool, L3 5QA, UK*

[4] *Research Centre for Drugs and Diagnostics, Liverpool School of Tropical Medicine, Liverpool, L3 5QA, UK*

S1.1 Plasmin bioassay development and optimization

The plasmin bioassay is a fluorescence-based bioassay in which conversion of the fluorogenic substrate H-D-Val-Leu-Lys-AMC by plasmin to its fluorescent product 7-amino-4-methylcoumarin is measured. The bioassay developed for this study is based on the protocol published by Tervo et al.[1]

The optimization procedure included selection of the concentration and pH of the buffer, the concentration of plasmin and of H-D-Val-Leu-Lys-AMC, and optimization of the well volume. A Tris-HCl buffer was chosen and tested at 50 and 100 mM, and at pH 7.0, 7.5, 7.8 and 8.0. For these experiments, the enzyme and the substrate were diluted in the respective buffers to final concentrations of 100 ng/ml and 5 µM, respectively. The measurements were replicated six times using black, 384-well format, microtiter well plates. The fluorescence was measured kinetically with a VarioSkan LUX microplate reader set to an excitation and emission wavelength of 380 and 460 nm, respectively, with a bandwidth of 12 nm. The measurements were performed at 37 °C. The 100 mM buffer (pH 7.5) showed the best performance (i.e. showing the smallest signal variation between replicates and the highest fluorescence signal intensity) (Figures S3a and S3b for 100 mM and 50 mM, respectively).

Using the optimized buffer, ten H-D-Val-Leu-Lys-AMC concentrations between 0 and 50 µM were tested at an enzyme concentrations of 100 ng/ml. Substrate at each concentration was mixed with plasmin, and the product formation was measured in time. For each well, an enzymatic reaction curve was generated and all curves were then used to calculate K_M value (31 µM) using the Michaelis-Menten kinetic model using Prism v.5 software (Figure S3c). At 5.0 µM substrate a stable baseline with a satisfying S/N ratio was obtained. Considering the high price of the substrate, 5 µM substrate was chosen for the bioassay.

For selection of the optimal assay volume, the bioassay mixture was tested at 25, 50 and 75 µL volumes. For each tested volume, bioassay mixture was pipetted into 300 wells of a black 384-well microtiter plate using a Multidrop 384 reagent dispenser. The enzymatic activity was measured over 10 cycles. The slope for each well over these 10 cycles was calculated and plotted against the well number. The readout of the well plate was performed in a serpentine way (from well 1A to 1K, and then from 2K to 2B, etc. covering the whole 384 well plate). This readout fashion will mimic the readout of a nanofractionated plate, also performed in a serpentine way. The data was plotted as if it was a “blank” nanofractionated plate for which the signal should be the same in all wells. This way discrepancies can now

nicely be observed since edge effects are observed as regular zigzag patterns in the plotted data. From these results the optimal mixture volume (i.e. the data showing the least differences in highest and lowest signals, and the smallest zigzag pattern) was determined to be 50 µL (Figure S3d).

The fully optimized plasmin bioassay mixture (50 µL) comprised 100 ng/ml plasmin and 5 µM substrate in 100 mM Tris-HCl buffer (pH 7.5) with 0.1% BSA.

S1.2 NanoLC-MS/MS analysis of tryptic digests

The nanoLC system consisted of a NCS-3500RS Nano/Cap System with a NCP-3200RS Nano/Cap Pump and WPS-3000(RS) Autosampler UltiMate 3000 Series from Thermo Scientific. Prior to separation, samples were pre-concentrated on a C18 trapping column (Acclaim™ PepMap™ 100 C18 LC Columns, 5µm, 5mm length, 0.3mm I.D. from Thermo Fisher Scientific) connected to the analytical column (Acclaim™ PepMap™ 100 C18 LC Columns, 2µm, 150mm length, 0.75mm I.D. from Thermo Fisher Scientific) via a 6-port switch valve. Both columns were kept at 45 °C. Analyses started by injecting 1 µL of sample onto the trapping column via a 20 µL injection loop (the injection mode used was µL-pickup, in which sample is aspirated with help of a transport liquid, which was pure water) at a flow rate of 10 µL/min using the sample loading pump. At this stage, the trapping column was not in line with the nanoflow solvent (NS) pumps. After 4 min the 6-port valve was switched to the position in which the pre-column was in line with the NS pumps enabling the trapped peptides and proteins to be eluted onto the analytical column. The flow rate of the NC pump was maintained at 0.5 µL/min throughout the analysis. Separation was performed using binary gradient elution with solvent A comprising water with 0.1% FA (v/v) and solvent B comprising 80% ACN, 20% water and 0.1% FA (v/v/v). The following gradient was used for separation (after trapping 4 min): from 4-10 min an isocratic flow at 1% B, which increased to 20% B in 10-15 min. From 15-45 min, the percentage B increased to 45% and next within 1 min it increased to 85% B, where it remained for 5 min. Then in 0.5 min it returned to initial conditions. Column equilibration time was set at 8.5 min. At 59 min, the 6-port valve was switched back to the position in which the trapping column was in line with the loading pump delivery system for the next 1 min. The nanoLC system was controlled by Chromeleon version 7.2 SR4 software. A Maxis HD quadrupole time-of-flight MS (Bruker Daltonics, Bremen, Germany) was operated in positive electrospray ionization (ESI) mode with a capillary voltage set at 1.3-1.4 kV, a dry gas flow rate of 3 l/min and dry gas temperature set

at 150 °C. Analyses were performed using auto MS/MS scan mode. Full mass spectra were acquired (*m/z* 500-2000), from which the three most intense ions per scan were fragmented using collision induced dissociation (CID) in stepping mode. The parameters used for fragmentation were: collision energy, 80-100%; timing, 50%. Parameters used for the basic stepping can be found in Table S1. Mass accuracy of 1-2 ppm was ensured by calibrating the system with an ESI-L Low concentration Tuning Mix (Agilent, Santa Clara, California, USA).

S2.1 Detailed plasmin bioassay results of the six bioassay variants

Cb and *Dr* were investigated for the presence of proteinases and their possible calcium and/or zinc dependency. The two venoms (4 mg/ml each) were nanofractionated and next exposed to six different variants of the plasmin bioassay, which are described in detail in the main manuscript of this study (Section "Profiling plasmin inhibition and proteolytic activity in *Cb* and *Dr* venoms").

Starting with the first variant, in which no plasmin was used, the bioactivity chromatograms are shown in Figure 3b for *Cb* and *Dr*. For clarity, first the results of *Cb* venom and then these of *Dr* venom will be discussed. Considering that the bioactive peak in *Cb* venom did not disappear when running the bioassay without plasmin, most probably other proteases present in the venom are directly responsible for conversion of the substrate to its fluorescent product. Venom derived from *Cb* is known to be rich of proteolytic enzymes that show fibrino(geno)lytic properties. An example includes basilase, which is a metalloprotease containing zinc and calcium ions (1 and 2 mol per mol of protein, respectively), suggesting that its activity is dependent on both metal ions [2]. Assessment of the type of protease identified in the bioactivity chromatogram, i.e. whether it belongs to a family of metalloproteases or serine protease, was performed by exposing nanofractionated snake venom to the second and third variant of the bioassay mixture (i.e. without plasmin and with the addition of zinc (0.2 mM) or calcium (1 mM) ions, respectively). An increase of a bioactivity peak after addition of any of the two ions suggests dependence of the bioactive protein on the particular metal ion added. Zinc and calcium ions are known constituents of snake venom proteases, which are vital for the catalytic activity of metalloproteinases and also stabilize their structural conformation [3]. The results of the bioassays were compared to the ones in which only the substrate was used (Figure 3a). Figure 3d shows a threefold increase when the venom was exposed to a bioassay mixture enriched with calcium ions. The

addition of zinc ions, however, showed no significant changes in intensity of the bioactivity peak (Figure 3c).

Variation between plates can be expected as introduced by the chromatographic analyses, the time between placing venom in the cooled autosampler and actual analysis, the time between nanofractionation and vacuum centrifugation, the duration of vacuum centrifugation, as well as small changes in activity of the bioassay constituents during bioassay preparation. In order to assure that the variations observed in the bioassay are not a result of the variation between different plates/bioassays tested, additional experiments confirming the dependence of the bioactive compounds on the metal ions were performed. In these experiments two bioassay mixtures, one enriched with calcium ions and the other with zinc ions, were pipetted into alternating wells of one microtiter well plate containing nanofractionated venom. Here it has to be noted that the nanofractionation is done in serpentine fashion with filling the wells of columns of a well plate from left to right. Pipetting alternating wells with calcium or zinc solutions is done perpendicular to the nanofractionation pattern (i.e. alternating rows of a well plate are filled). This experiment confirmed a positive influence of calcium ions on the eluted bioactive proteins, as a twofold higher intensity of the bioactive peak was observed.

Finally, the presence of metalloproteases was further confirmed by an experiment in which the metal ion chelator 1,10-phenanthroline (5 mM) or EDTA (50 mM) was added to the bioassay mixture. Figure 3f shows a bioactivity chromatogram of *Cb* venom after 1,10-phenanthroline addition in which the bioactivity peak intensity did not change compared to the bioactivity chromatogram obtained from the experiment without the zinc chelator (Figure 3c). EDTA, which is known to chelate divalent ions including calcium and zinc, was observed to decrease the intensity of the activation peak by twofold (Figure 3e). The decrease of the peak intensity in this experiment indicated that proteases present in the *Cb* venom probably are calcium ion dependent. To reassure that the lower intensity of the bioactivity peak was indeed influenced by the addition of EDTA, and not by earlier mentioned variations observed between measurements, also here bioassay mixtures with and without EDTA were pipetted into alternating wells onto one microtiter well plate containing nanofractionated *Cb* venom. The results of this experiment (Figure S4b) confirmed that by removing calcium ions from the sample, the bioactivity decreased supporting the conclusion that indeed the positive peak observed was at least partly resulting from a calcium dependent proteolytic enzyme. Another explanation to the observed results could be that the positive peak contains two or more proteases, in which only one is calcium dependent. The removal of calcium ions would

cause a substantial decrease in the activity peak of the calcium dependent proteases, however not for other non-calcium dependent ones.

In case of *Dr* venom, the use of bioassay mixture without plasmin addition (first variant of the bioassay mixture) resulted in disappearance of the negative peaks, as plasmin was not present in the bioassay mixture, and no presence of activation peaks was observed either (Figure 3b). The addition of zinc ions (variant 2) and addition of calcium ions (variant 3, Figure 3d) to the bioassay mixture not containing plasmin showed no bioactivity. A further experiment in which EDTA (variant 5) was added to the bioassay mixture containing only the substrate, showed no bioactivity (Figure 3e). Addition of 1,10-phenanthroline to the mixture containing the enzyme and substrate revealed two additional negative peaks between 14 and 18 min in the bioactivity chromatogram (Figure 3f). These negative peaks might represent additional plasmin inhibitors, which due to a possible co-elution with metal dependent proteases that were not detected possibly due to their low concertation or low stability, could not be identified after exposing the nanofractionated venom to the normal variant of the bioassay (i.e. only containing plasmin and the substrate). Therefore, the two negative peaks most probably are plasmin inhibitors.

References:

1. Tervo T, Honkanen N, Setten G-B van, Virtanen T, Tarkkanen A, Harkonen M (1994) A rapid fluorometric assay for tear fluid plasmin activity. Cornea 13:148–155.

2. Datta G, Dong A, Witt J, Tu AT (1995) Biochemical Characterization of Basilase, a Fibrinolytic Enzyme from Crotalus basiliscus basiliscus. Arch Biochem Biophys 317:365–373.

3. Takeya H, Nishida S, Nishino N, Makinose Y, Omori-Satoh T, Nikai T, Sugihara H, Iwanaga S (1993) Primary structures of platelet aggregation inhibitors (disintegrins) autoproteolytically released from snake venom hemorrhagic metalloproteinases and new fluorogenic peptide substrates for these enzymes. J Biochem 113:473–483.

Figures Supplementary Materials

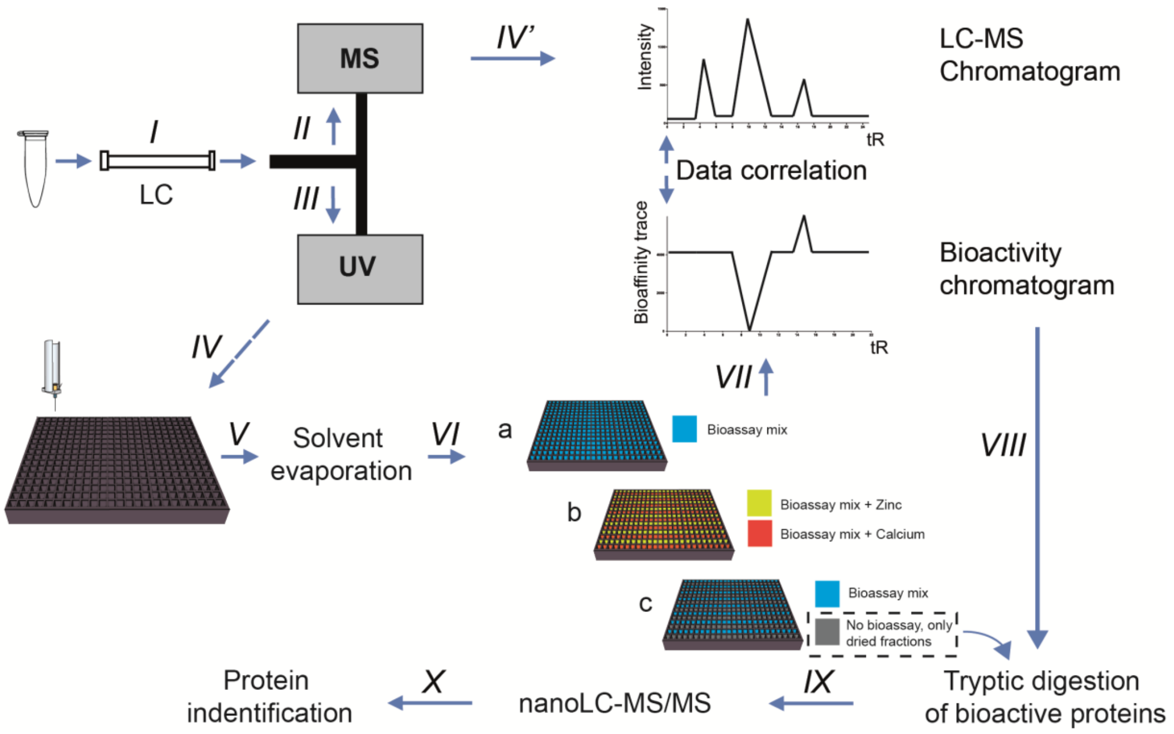

Fig. S1 Nanofractionation system with parallel MS data acquisition. After nanofractionation and vacuum centrifuge drying of the 384-well plate, a fluorescence plasmin activity assay is performed. (*I*) Chromatographic separation of snake venom; (*II*) Split (10% of the column effluent) to MS analysis; (*III*); UV detection followed by (*IV*) high resolution (6 s) nanofractionation of 90% of the column effluent; (*IV'*) MS analysis; (*V*) Evaporation of solvent; (*VI*) Bioassay mixture addition: a) addition of the full bioassay mixture containing the enzyme and the substrate, b) addition of the bioassay mixture enriched with zinc ions (yellow) or calcium ions (red) in alternative way; c) addition of the full bioassay mixture to every second well of the well plate; (*VII*) Fluorescence readout; (*VIII*) Selection of wells with bioactive proteins for tryptic digestion; (*IX*) Analysis of tryptic digests using nanoLC-MS/MS; (*X*) Identification of bioactive proteins using Mascot database search

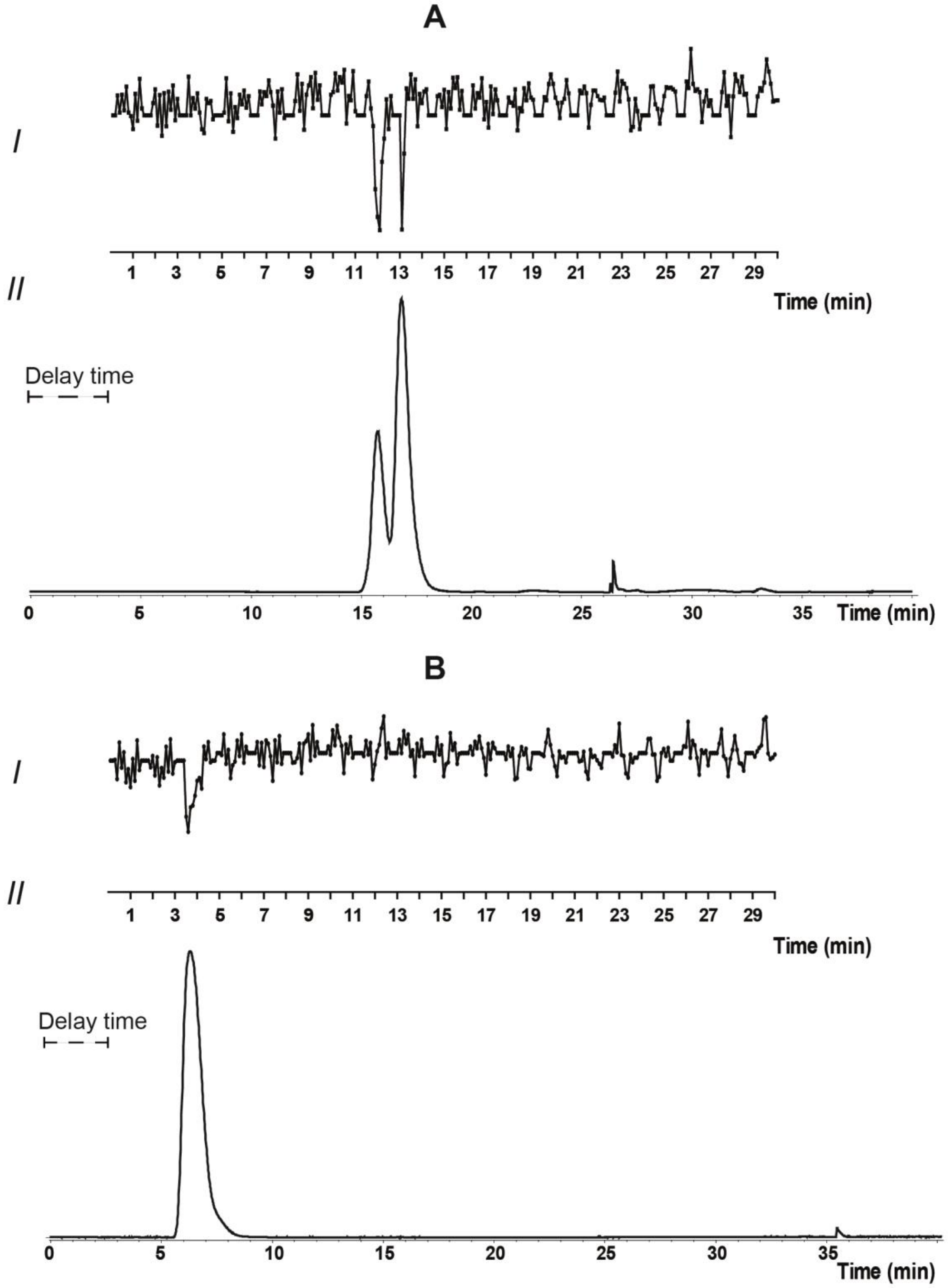

Fig. S2 Time alignment of the nanofractionated bioassay and the MS data using leupeptin for (A) RPLC analysis, and (B) HILIC analysis. *I,* bioactivity chromatograms; *II*, extracted ion chromatograms. Leupeptin $[M+H]^+$ is 427.3025 Da.

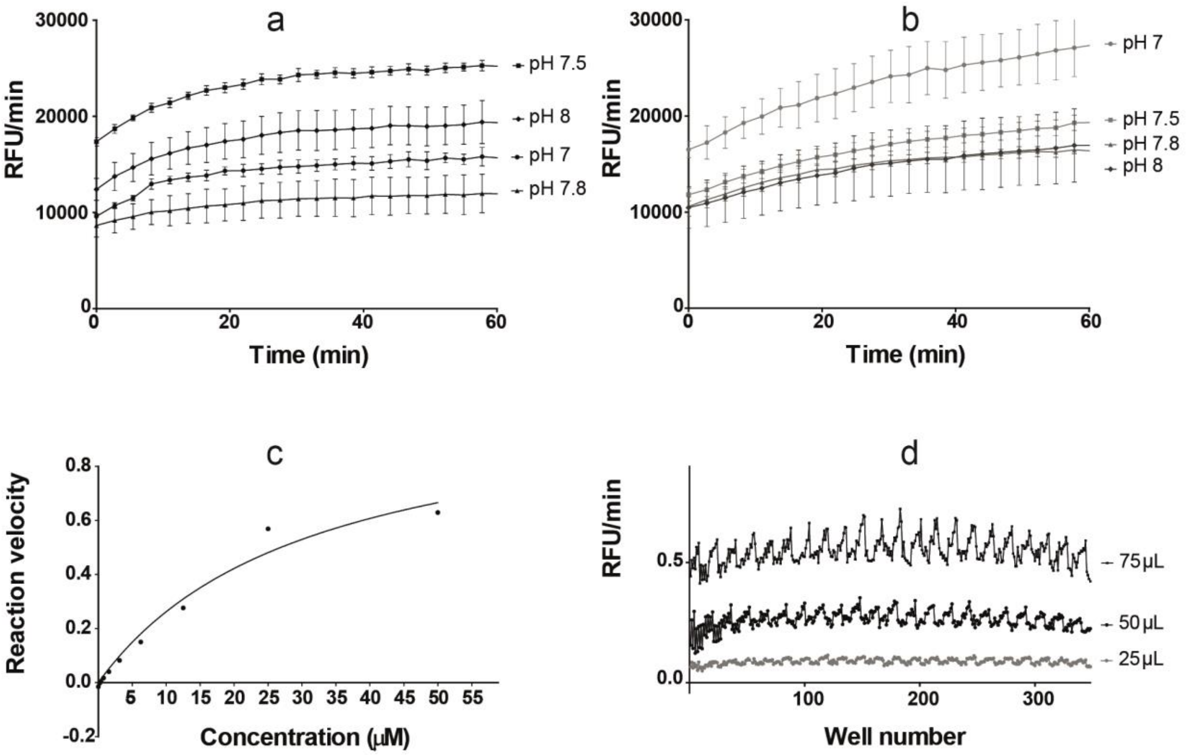

Fig. S3 Bioassay optimization: (a) 50 mM TRIS-HCl buffer at various pH; and (b) 100 mM TRIS-HCl buffer at various pH; (c) Determination of K_M value at 100 ng/ml plasmin and ten substrate concentrations ranging between 0 and 50 µM. (d) Bioassay mixture volume optimization using 100 ng/ml plasmin and 5 µM substrate in 0.1 M TRIS-HCl buffer with 0.1 % BSA

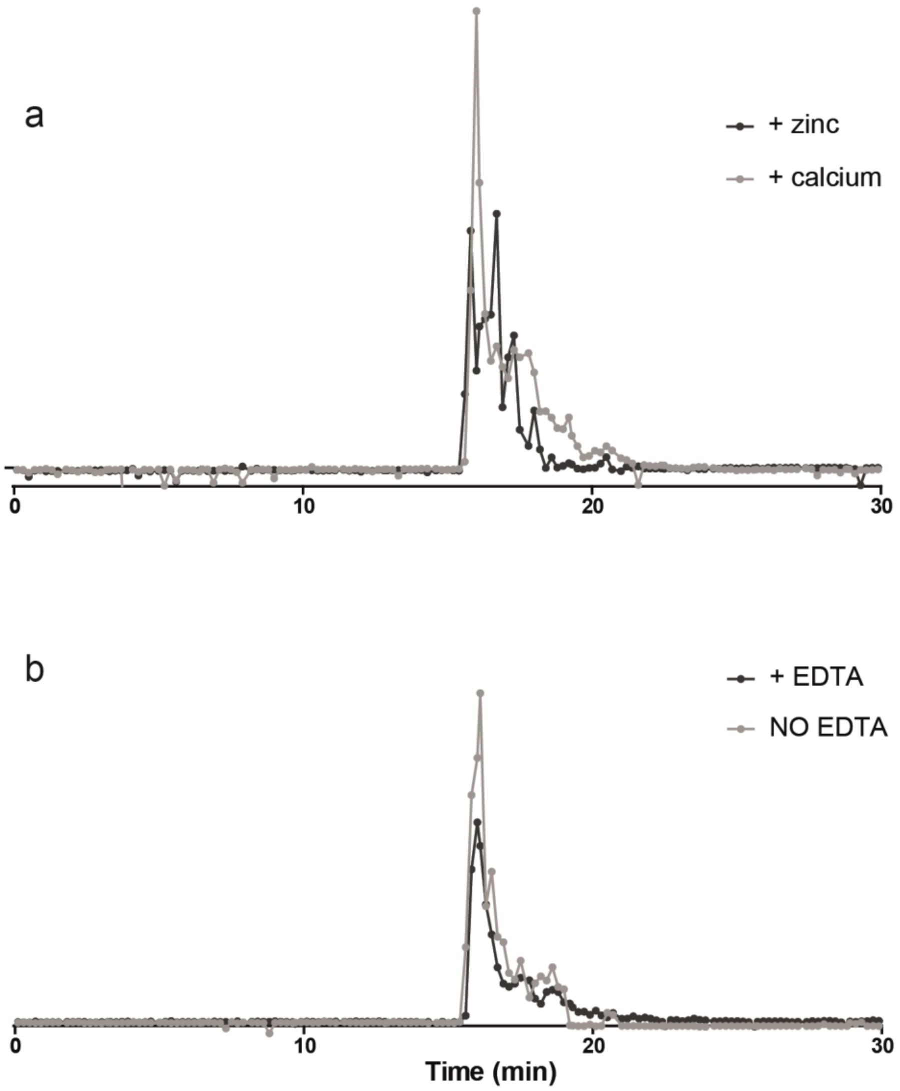

Fig. S4 Bioassay chromatograms of *Cb* venom nanofractionated on a single plate and exposed to two different bioassay mixtures using the alternating well method. (a) Plasmin bioassay with alternating zinc/calcium enrichment; (b) Plasmin bioassay with alternating EDTA/no EDTA addition

Table S1 Isolation and fragmentation parameters used for nanoLC-MS/MS analysis of the digested bioactive proteins and peptides found in *Cb* and *Dr*.

Type	Mass	Width	Collision	Charge state
**Base**	500	8	35	1
**Base**	500	8	30	2
**Base**	500	8	25	3
**Base**	500	8	20	4
**Base**	1000	10	50	1
**Base**	1000	10	40	2
**Base**	1000	10	35	3
**Base**	1000	10	30	4
**Base**	2000	15	70	1
**Base**	2000	15	50	2
**Base**	2000	15	45	3
**Base**	2000	15	40	4

Table S2 Mascot search results for *Cb* bioactives. The information in the table includes: The accession number, protein score, protein name, protein % coverage, number of matches, Mr Obs (observed molecular mass), Mr Exp (expected molecular mass, Mr Calc (calculated molecular mass), peptide score, and Expect (expectation value).

***Crotalus basiliscus* Hilic**											
**Accession number**	**Protein score**	**Protein name**	**% Coverage**	**No of matches**	**Number of distinct sequences**	**Mr Obs.**	**Mr Exp.**	**Mr Calc.**	**Peptide Score**	**Expct.**	**Peptide sequence**
VSP_CRODD	163	Snake venom serine protease OS=Crotalus durissus durissus PE=2 SV=1	17	7	3	559.7972	1117.5799	1117.5801	70	0.00098	R.TLCAGILEGGK.G
						1249.5829	2497.1512	2497.1472	63	7.30E-05	K.VFDYTEWIQSIIAGNTDATCPP.-
						504.9026	1511.6858	1511.6787	44	4.00E-03	L.VIGGDECNINEHR.S
VSP1_CROAT	129	Snake venom serine protease catroxase-1 OS=Crotalus atrox PE=1 SV=1	24	6	4	559.7972	1117.5799	1117.5801	70	0.00098	R.TLCAGILEGGK.D
						698.348	1394.6814	1394.683	61	0.004	R.AAYPEYGLPATSR.T
						715.358	1428.7014	1428.7031	73	0.00016	L.SLPSSPPSVGSVCR.I
						960.792	2879.3543	2879.3589	48	0.00015	K.VFDHLDWIQSIIAGNTDATCPFVNF.-
VSP2_CROAT	82	Snake venom serine protease catroxase-2 OS=Crotalus atrox PE=1 SV=1	12	2	2	500.2303	1497.6691	1497.663	55	0.0002	L.VVGGDECNINEHR.S
						714.7009	2141.0808	2141.0463	61	0.00027	K.LLDDAVCQPPYPELPATSR.T
VSPY_MACLB	71	Chymotrypsin-like protease VLCTLP OS=Macrovipera lebetine PE=1 SV=1	13	4	2	500.2303	1497.6691	1497.663	55	0.0002	L.VVGGDECNINEHR.S
						824.0618	2469.1637	2471.1679	39	0.0077	K.VFDYSDWIQSIIAGNTTATCPL.-

WM1AD_CROAT	123	Snake venom metalloproteinase atrolysin-D PE=1 SV=3	3	7	1	536.6153	1606.824	1606.8256	61	0.0005	R.VHEIVNFINGFYR.S
VSP3_PROJR	115	Snake venom serine protease 3 OS=Protobothrops jerdonii PE=2 SV=1	8	5	1	1242.0646	2482.1147	2482.1475	65	1.40E-05	N.VFNYTDWIQSIIAGNTDATCPP.-
PA2AF_CROVV	98	Acidic phospholipase A2 Cvv-E6f OS=Crotalus viridis viridis PE=1 SV=1	26	4	3	722.3081	1442.6017	1442.5965	53	0.0002	R.SGLFWYGAYGCY.C
						565.2405	1692.6996	1691.7131	53	0.0045	K.NGEIICEDDDPCKK.Q
						502.5192	1504.5357	1504.5356	35	0.0044	R.CCFVHDCCYGK.A
VM2I_CROBA	77	Disintegrin basilicin OS=Crotalus basiliscus PE=1 SV=1	23	1	1	684.2932	2049.8579	2049.8601	77	1.90E-06	K.LRPGAQCAEGLCCDQCR.F
***Crotalus basiliscus* RP C18**											
VSP_CRODD	233	Snake venom serine protease OS=Crotalus durissus durissus PE=2 SV=1	13	8	2	1249.5779	2497.1413	2497.1472	84	1.70E-07	K.VFDYTEWIQSIIAGNTDATCPP.-
						504.8994	1511.6763	1511.6787	47	0.0025	L.VIGGDECNINEHR.S
VSP1_CROAT	99	Snake venom serine protease catroxase-1 OS=Crotalus atrox PE=1 SV=1	10	2	2	715.3554	1428.6961	1428.7031	76	6.20E-05	L.SLPSSPPSVGSVCR.I
						698.346	1394.6775	1394.683	73	0.00061	R.AAYPEYGLPATSR.T
VSP2_CROAT	91	Snake venom serine protease catroxase-2 OS=Crotalus atrox PE=1 SV=1	17	3	3	739.8746	1477.7346	1477.7413	45	0.016	K.LDSPVSNSEHIAPL.S
						500.2308	1497.6707	1497.663	40	0.0036	L.VVGGDECNINEHR.S
						714.6984	2141.0735	2141.0463	74	6.90E-06	K.LLDDAVCQPPYPELPATSR.T
VSP3_PROJR	100	Snake venom serine protease 3 OS=Protobothrops jerdonii PE=2 SV=1	8	4	1	1242.057	2482.0994	2482.1475	52	0.00014	N.VFNYTDWIQSIIAGNTDATCPP.-

PA2AF_CROVV	59	Acidic phospholipase A2 Cvv-E6f OS=Crotalus viridis viridis PE=1 SV=1	18	2	2	502.5176	1504.5309	1504.5356	39	0.0028	R.CCFVHDCCYGK.A
						564.9099	1691.7078	1691.7131	51	0.0013	K.NGEIICEDDDPCKK.Q

Table S3 Mascot search results for *Dr* bioactives. The information in the table includes: The accession number, protein score, protein name, protein % coverage, number of matches, Mr Obs (observed molecular mass), Mr Exp (expected molecular mass, Mr Calc (calculated molecular mass), peptide score, and Expect (expectation value).

***Daboia russelii* HILIC**											
**Accession number**	**Protein score**	**Protein name**	**% Coverage**	**No of matches**	**Number of distinct sequences**	**Mr Obs.**	**Mr Exp.**	**Mr calc.**	**Score**	**Expect.**	**Peptide sequence**
PA2B8_DABRR	2173	Basic phospholipase A2 VRV-PL-VIIIa OS=Daboia russelii PE=1 SV=1	80	63	14	471.7264	941.4383	941.4317	47	0.036	L.YPDFLCK.G
						484.2488	966.4831	966.477	45	0.05	R.QNLNTYSK.K
						495.2599	988.5053	988.5012	69	0.00025	R.VNGAIVCEK.G
						675.3211	1348.6277	1348.6196	68	0.00018	K.YMLYPDFLCK.G
						683.3174	1364.6203	1364.6145	47	0.029	K.YMLYPDFLCK.G + Oxidation (M)
						691.2724	1380.5302	1380.5227	75	6.80E-06	Y.SSYGCYCGWGGK.G
						729.3143	1456.614	1456.6149	41	9.10E-03	R.ICECDKAAAICF.R
						538.5818	1612.7236	1612.716	73	5.30E-06	R.ICECDKAAAICFR.Q
						892.3818	1782.7491	1782.7382	74	2.60E-03	K.LAIPSYSSYGCYCGW.G
						897.4021	1792.7897	1792.8196	109	9.20E-09	R.VNGAIVCEKGTSCENR.I
						598.6039	1792.7899	1792.8196	77	8.60E-07	R.VNGAIVCEKGTSCENR.I
						646.3085	1935.9038	1935.8933	46	1.90E-03	K.YMLYPDFLCKGELKC.-
						650.6381	1948.8923	1948.9207	83	3.10E-07	K.RVNGAIVCEKGTSCENR.I
						675.9692	2024.8857	2024.8761	82	3.00E-07	K.LAIPSYSSYGCYCGWGGK.G
						1013.4503	2024.8861	2024.8761	112	8.90E-10	K.LAIPSYSSYGCYCGWGGK.G

						772.6319	2314.8738	2314.8687	73	1.10E-05	R.CCFVHDCCYGNLPDCNPK.S
						975.7852	2924.3337	2926.334	97	3.80E-09	K.MILEETGKLAIPSYSSYGCYCGWGGK.G
PA2B5_DABRR	1935	Basic phospholipase A2 VRV-PL-V OS=Daboia russelii PE=1 SV=1	71	62	16	471.7264	941.4383	941.4317	47	0.036	L.YPDFLCK.G
						484.2488	966.4831	966.5134	45	0.05	T.KNLNTYSK.I
						495.2599	988.5053	988.4648	69	0.00025	R.VNGAIVCEQ.G
						675.3211	1348.6277	1348.6196	68	0.00018	I.YMLYPDFLCK.G
						683.3174	1364.6203	1364.6145	47	0.029	I.YMLYPDFLCK.G + Oxidation (M)
						691.2724	1380.5302	1380.5227	75	6.80E-06	Y.SSYGCYCGWGGK.G
						713.8253	1425.636	1425.6275	36	0.0024	K.LAVPFYSSYGCY.C
						729.3143	1456.614	1456.6149	41	0.0091	R.ICECDKAAAICF.T
						731.8638	1461.7131	1461.7036	59	0.0013	K.IYMLYPDFLCK.G
						739.8609	1477.7072	1477.6985	74	0.00015	K.IYMLYPDFLCK.G + Oxidation (M)
						849.4331	1696.8516	1696.8416	117	3.60E-09	-.SLLEFGMMILEETGK.L
						857.4293	1712.844	1712.8365	104	3.90E-08	-.SLLEFGMMILEETGK.L + Oxidation (M)
						857.4301	1712.8456	1712.8365	110	1.40E-08	-.SLLEFGMMILEETGK.L + Oxidation (M)
						865.4265	1728.8385	1728.8314	73	1.20E-05	-.SLLEFGMMILEETGK.L + 2 Oxidation (M)
						897.4021	1792.7897	1792.	109	9.20E-09	R.VNGAIVCEQGT

								7832			SCENR.I
						598.6039	1792.7899	1792.7832	77	8.60E-07	R.VNGAIVCEQGTSCENR.I
						915.3926	1828.7707	1828.7589	72	1.10E-06	K.LAVPFYSSYGCYCGW.G
						630.6594	1888.9563	1888.9467	47	0.0018	K.IYMLYPDFLCKGELK.C
						646.3085	1935.9038	1935.8933	46	0.0019	I.YMLYPDFLCKGELKC.-
						650.6381	1948.8923	1948.8843	83	3.10E-07	K.RVNGAIVCEQGTSCENR.I
						691.3089	2070.9048	2070.8968	66	0.0028	K.LAVPFYSSYGCYCGWGGK.G
						1036.4617	2070.9089	2070.8968	91	1.30E-05	K.LAVPFYSSYGCYCGWGGK.G
						1064.9719	2127.9292	2127.9183	28	0.024	K.LAVPFYSSYGCYCGWGGK
PA2B_VIPRE	1463	Basic phospholipase A2 vurtoxin OS=Vipera renardi PE=1 SV=1	29	35	4	471.7264	941.4383	941.4317	47	0.036	Y.YPDFLCK.K
						849.4331	1696.8516	1696.8416	117	3.60E-09	G.SLLEFGMMILEETGK.N
						857.4293	1712.844	1712.8365	104	3.90E-08	G.SLLEFGMMILEETGK.N + Oxidation (M)
						857.4301	1712.8456	1712.8365	110	1.40E-08	G.SLLEFGMMILEETGK.N + Oxidation (M)
						865.4265	1728.8385	1728.8314	73	1.20E-05	G.SLLEFGMMILEETGK.N + 2 Oxidation (M)
						877.9431	1753.8716	1753.863	95	1.70E-07	E.GSLLEFGMMILEETGK.N
						772.6319	2314.8738	2314.8687	73	1.10E-05	R.CCFVHDCCYGNLPDCNPK.I
VKT3_DABRR	65	Kunitz-type serine protease inhibitor 3 OS=Daboia russelii PE=2 SV=1	20	1	1	651.9631	1952.8674	1952.8588	65	0.0063	K.EFIYGGCHGNANNFPTR.D
PA2AI_TRIST	63	Acidic phospholipase A2 CTs-A3 OS=Trimeresurus stejnegeri PE=1 SV=1	11	1	1	557.5885	1669.7437	1669.7375	63	0.00017	K.AVCECDKAAAICFR.D

Daboia russelii **RP C18**											
PA2B_VIPRE	493	Basic phospholipase A2 vurtoxin OS=Vipera renardi PE=1 SV=1	11	11	2	849.4249	1696.835 2	1696. 8416	94	1.60E-07	G.SLLEFGMMILE ETGK.N
						877.9387	1753.862 7	1753. 863	96	2.40E-07	E.GSLLEFGMMIL EETGK.N
VKTC6_DABSI	343	Kunitz-type serine protease inhibitor C6 OS=Daboia siamensis PE=2 SV=1	43	8	5	677.2835	1352.552 5	1352. 5528	66	0.00029	Y.GGCGGNANNF ETR.D
						798.3331	1594.651 6	1594. 6511	78	4.00E-07	R.FYYNPASNQCQ GF.T
						367.1814	1464.696 3	1464. 7004	32	0.03	R.HTCGASGNVGP RPR.I
						809.3397	1616.664 8	1616. 6638	140	3.30E-12	F.TYGGCGGNAN NFETR.D
						1065.442	3193.304 1	3193. 3043	128	2.20E-12	R.FYYNPASNQCQ GFTYGGCGGNAN NFETR.D
VKT4_DABRR	125	Kunitz-type serine protease inhibitor 4 OS=Daboia russelii PE=2 SV=1	33	3	3	677.2835	1352.552 5	1352. 5528	66	0.00029	Y.GGCGGNANNF ETR.D
						798.3331	1594.651 6	1594. 6511	78	4.00E-07	R.FYYNPASNQCQ GF.I
						814.8215	1627.628 3	1628. 7001	45	0.00079	F.IYGGCGGNANN FETR.D
VKT3_DABRR	58	Kunitz-type serine protease inhibitor 3 OS=Daboia russelii PE=2 SV=1	36	4	2	855.3362	1708.65 78	1708. 6572	33	0.02	K.FCYLPADPGE CMAY.I + Oxidation (M)
